# A universal model of electrochemical safety limits *in vivo* for electrophysiological stimulation

**DOI:** 10.3389/fnins.2022.972252

**Published:** 2022-10-06

**Authors:** Ritwik Vatsyayan, Shadi A. Dayeh

**Affiliations:** Integrated Electronics and Biointerfaces Laboratory, Department of Electrical and Computer Engineering, University of California, San Diego, San Diego, CA, United States

**Keywords:** stimulation, pulse width, impedance, safety limit, electrophysiology

## Abstract

Electrophysiological stimulation has been widely adopted for clinical diagnostic and therapeutic treatments for modulation of neuronal activity. Safety is a primary concern in an interventional design leveraging the effects of electrical charge injection into tissue in the proximity of target neurons. While modalities of tissue damage during stimulation have been extensively investigated for specific electrode geometries and stimulation paradigms, a comprehensive model that can predict the electrochemical safety limits *in vivo* doesn’t yet exist. Here we develop a model that accounts for the electrode geometry, inter-electrode separation, material, and stimulation paradigm in predicting safe current injection limits. We performed a parametric investigation of the stimulation limits in both benchtop and *in vivo* setups for flexible microelectrode arrays with low impedance, high geometric surface area platinum nanorods and PEDOT:PSS, and higher impedance, planar platinum contacts. We benchmark our findings against standard clinical electrocorticography and depth electrodes. Using four, three and two contact electrochemical impedance measurements and comprehensive circuit models derived from these measurements, we developed a more accurate, clinically relevant and predictive model for the electrochemical interface potential. For each electrode configuration, we experimentally determined the geometric correction factors that dictate geometry-enforced current spreading effects. We also determined the electrolysis window from cyclic-voltammetry measurements which allowed us to calculate stimulation current safety limits from voltage transient measurements. From parametric benchtop electrochemical measurements and analyses for different electrode types, we created a predictive equation for the cathodal excitation measured at the electrode interface as a function of the electrode dimensions, geometric factor, material and stimulation paradigm. We validated the accuracy of our equation *in vivo* and compared the experimentally determined safety limits to clinically used stimulation protocols. Our new model overcomes the design limitations of Shannon’s equation and applies to macro- and micro-electrodes at different density or separation of contacts, captures the breakdown of charge-density based approaches at long stimulation pulse widths, and invokes appropriate power exponents to current, pulse width, and material/electrode-dependent impedance.

## Introduction

The clinical use of pulsed electrophysiological stimulation for eliciting neuronal activity in the brain and spinal tissue has been widely adopted as both a diagnostic and therapeutic tool (Lozano et al., 2002; Vitek, 2002; Schwalb and Hamani, 2008; Boon et al., 2009; Herrington et al., 2016). Electrophysiological stimulation is also used in the operating room for neuromonitoring and mapping during surgical resections (Koulouris et al., 2012; Tchoe et al., 2022). Additionally, electrophysiological stimulation has been widely adopted as the cutting edge technology for treating neurodegenerative disorders by neuromodulation for Parkinson’s disease (Pollak et al., 2002; Benabid, 2003) and Alzheimer’s disease (Chang et al., 2018), as well as for neurological disorders such as obsessive-compulsive disorder (Nuttin et al., 1999; Jiménez et al., 2013), depression (Mayberg et al., 2005; Rao et al., 2018) and epilepsy (Theodore and Fisher, 2004; Fisher and Velasco, 2014).

Notwithstanding their ubiquitous presence in clinical treatment paradigms, there exists a need for systematic studies investigating the underlying device constraints and charge transfer mechanisms for determining safe stimulation levels. The Shannon’s equation (Shannon, 1992) has been widely adopted to empirically determine the tissue damage thresholds for electrophysiological stimulation *in vivo* by setting empirical limits that relate the injected charge density during stimulation to the charge injected per phase. However, the Shannon equation does not account for several parameters including the pulse width, stimulation setup, electrode geometry, contact material and the electrochemical interface at the stimulating contact (Cogan, 2006; Cogan et al., 2016). Subsequent studies have looked into the role of stimulation parameters such as frequency of the stimulation pulses (McCreery et al., 1995, 1997; Butterwick et al., 2007) and the electrochemical interface (Cogan, 2008), but have stopped short of predicting a generalized model that can be applied across stimulation modalities. Empirical studies of tissue damage thresholds during electrical stimulation led to the establishment of other commonly used safety limits, such as the 30 μC/cm^2^ limit for stimulation from macro contacts (McCreery et al., 1990) and the 4nC/phase limit for stimulation from micro contacts (McCreery et al., 2002). Further, the difference in electrochemical safety limits obtained from benchtop settings and from *in vivo* measurements has not been sufficiently explored (Han et al., 2012). We hypothesized that detailed benchtop and *in vivo* characterization across different length scales of contact diameter and separation and for different contact materials and across a wide space of stimulation parameters may lead to a universal equation that can capture the electrochemical safety limits. We carried out these studies and developed such an equation in this work.

In addition to the absence of a comprehensive experimental paradigm for determining safety limits, there is a disconnect between typical electrochemical characterization techniques and practical electrophysiological stimulation setups. Conventionally, Electrochemical Impedance Spectroscopy (EIS) is performed in benchtop setups to characterize the electrode-electrolyte interface with a Ag/AgCl reference electrode and a low impedance counter electrode (typically a Pt wire) (Merrill et al., 2005). However, most conventional stimulation paradigms are bipolar in nature- that is the reference and counter electrodes are shared on a single contact (Meyer et al., 2001). The absence of the third electrode means that both the injecting and the reference electrodes now play a role in the voltage drop at either interface and consequently, they impact the observed electrolysis window and electrochemical safety limits. Thus, it is critical to examine each element of the electrode-medium interface independently and examine their role in establishing the stimulation safety limits. To determine the current pathway and voltage drops across each of these elements, one must perform impedance spectroscopy in 2-, 3-, and 4-contact configurations to delineate the electrochemical interface impedance from the series impedance elements, including the medium impedance and the resistance due to the circuit connections between the measurement system and the electrode under analysis. These impedances need to be validated in both benchtop and *in vivo* setups to establish the safety limits (Grill and Mortimer, 1995).

Tissue damage in clinical stimulation can occur due to different mechanisms that include the electrochemical generation of irreversible and harmful reaction by products (Bullara et al., 1988), mechanical implantation damage or physiological response from the body (Somann et al., 2018; Straka et al., 2018; Seaton et al., 2020). Further, it has been previously reported that the impedance spectra of an electrode can show significant variations depending on the medium the electrode is placed in - that is tissue or saline (Wei and Grill, 2009; Alba et al., 2015; Vatsyayan et al., 2021). Further, the electrode impedance is also known to vary with time post-implantation in *in vivo* chronic setups (Prasad and Sanchez, 2012; Black et al., 2018). Thus, a singular limit for stimulation safety appears incomplete to deal with the multiple stimulation paradigms commonly used in research and clinical practice.

**FIGURE 1:**
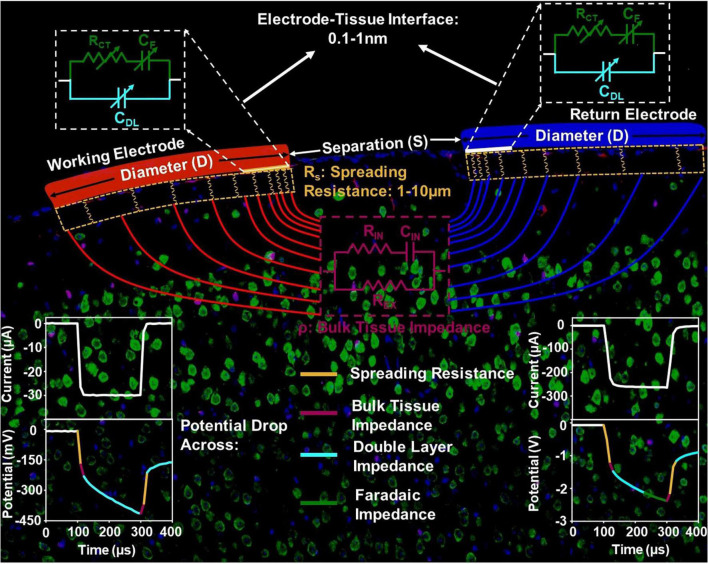
The circuit model of the current injection mechanism *in vivo*, with the impedance elements corresponding to the electrode-tissue interface, spreading resistance and bulk tissue resistance spatially delineated.

To establish this limit, we first overview the details of elements involved in current injection into and across the biological tissue to frame our analysis. Figure 1 shows the setup for bipolar *in vivo* stimulation. Current is injected and extracted from the tissue through identical electrode contacts of diameter *D*, and Separation *S*. There are three main components in the current flow. The electrode-tissue interface, at both the working (injecting) and counter (extracting) contact, forms the capacitive network for charge injection from the contact into the surrounding tissue. The electrochemical interface is composed of a very thin layer of ions with a proximity of about 0.1-1nm from the surface of the contact where free charge carriers (electrons) reside (Bohinc et al., 2001; Ruzanov et al., 2018). Therefore, near equilibrium, the capacitive charge screening element of the electrode tissue interface is modeled with a constant phase-element component, that is a non-ideal double-layer capacitor, *C*_*DL*_, whose impedance, *Z*_*DL*_, has a slightly weaker dependence on frequency compared to ideal capacitors. Current across the interface can also be carried by direct charge transfer between the electrode and tissue and can be modeled by a resistive charge transfer element, *R*_*CT*_, and another constant phase-element, *C*_*F*_, that captures the direct charge transfer to and from ions that can migrate away but not far from the double-layer and can participate in a redox reaction. Charge transfer between the electrode and medium is governed by an energy barrier whose magnitude is determined by the difference between the Fermi Energy in the contact and the electrochemical potential in tissue. The Fermi energy is the energy at which the probability of finding an occupied electronic state in a solid-state material (e.g., a metal contact electrode) is exactly one half. The electrochemical potential is an energy level at which the probability of finding an occupied electronic state and a negatively charged ion, in a mixed electronic-ionic medium (e.g., saline or tissue), is exactly one half. Therefore, both energy levels are determined by the respective concentration of charge carriers. With a potential bias to inject current, or with injected current that gives rise to a potential buildup between the contact and tissue, this energy barrier is overcome for direct charge transfer. The higher the applied potential at the electrode-tissue interface, the more efficient the direct charge transfer. As a result, all elements of the electrode-tissue interface are bias dependent and their value under stimulation can be very different from near equilibrium, the regime where electrochemical impedance spectroscopy is typically conducted.

The potential and the current in the tissue is directional between the two contacts because of which, current crowding occurs in a small volume of tissue next to the edge of the current injecting and extracting contacts. This effect is more pronounced for the contacts with smaller diameter. This current crowding gives rise to a spreading resistance in the surrounding tissue with a length scale of several micrometers. Therefore, the geometry of the electrode and the spatial position of the counter contact in relation to the working contact directly impact the observed spreading resistance. Further away from the contact, the current is conducted in the bulk of the tissue and can be modeled by volumetric conduction through a tissue impedance. The tissue impedance is composed of an extracellular resistance, *R*_*EX*_, that dominates the overall tissue conduction in the frequencies of interest for stimulation, as well as intracellular conduction that is modeled by an *R*_*IN*_ and *C*_*IN*_. Therefore, the potential drop across tissue is instantaneous (Figure 1).

With these length scales established, we can now isolate the potential of critical importance for electrochemical safety limits. The potential generated across the double-layer capacitance with a reactance *Z*_*DL*_ is the critical potential that determines whether electrolysis occurs in tissue and safety is compromised. For most contact materials, this potential can range from hundreds of mV to generally less than 2.5 V observed in our studies. Considering the thickness of the double layer capacitance to be 0.1–1 nm, the critical field ranges from approximately 1 V/1 nm to 1V/0.1 nm, or 10 MV/cm to 100 MV/cm. The spreading resistance usually leads to approximately 10 times the potential drop when compared to the potential drop across the double layer capacitance. Therefore, a crude estimation of the electric field within the tissue in the current crowding region where the spreading resistance arises, is 10 V/10 μm, which is 10 kV/cm, approximately 1000 times lower than that across the double-layer capacitance. This justifies the general practice in the calculation of charge injection capacity of neglecting instantaneous potential drops and the inspection of the potential build-up across the stimulation setup within the charge-injection phase. Lastly, the potential drop across tissue can be as small as 1/10*^th^* of the potential across the interface – when not accounting for gliosis which can raise the tissue impedance and therefore tissue potential further – and therefore has negligible influence on the electrochemical safety limits.

Here, we investigated the electrochemical safety limits for electrophysiological stimulation using three stimulation materials: platinum nanorods (PtNR), planar Pt and poly(3,4-ethylenedioxythiophene) polystyrene sulfonate (PEDOT:PSS, hereafter referred to as simply PEDOT). We leveraged our previous characterization of clinical electrocorticography (ECoG) and stereo-encephalography (sEEG) electrodes to validate the broader applicability of our model. We calculated the safety thresholds for each electrode in benchtop experiments by measuring the potential excursions generated on the electrode contact by the application of current excitations. To explore the contribution of the geometric design parameters of the electrode, we investigated the electrochemical safety limits for the platinum based electrodes (planar Pt and PtNR) over a wide range of electrode contact sizes, ranging from a diameter of 1mm down to a diameter of 20 μm. We changed the inter-contact separation for bipolar stimulation to study the effect of current crowding and the geometric correction factors on the electrophysiological stimulation and on the safety limits. We also investigated the influence of stimulation parameters by changing the pulse width. We then created an equation to predict the built-up potentials on the electrode interface as a function of these parameters. Finally, we validated the applicability of our model *in vivo* in acute measurements in the rat’s brain for the planar Pt and PtNR electrodes and in the pig’s brain for the surface ECoG, and clinical depth and sEEG electrodes. We benchmarked our calculated safety limits to the previously established safety limits to provide guidelines for establishing safe clinical stimulation paradigms.

## Experimental setup

To perform the electrochemical characterization of the electrodes in benchtop and *in vivo*, we used the Interface 1000E and 620 Potentiostats from Gamry Instruments. Our electrodes are built on thin-film parylene C for all surface grids (Ganji et al., 2017, 2019; Paulk et al., 2021; Yang et al., 2021), and we leveraged our prior characterization of clinical depth and surface grids to validate the broader applicability of our model. Our surface electrodes were fabricated on a 7 μm thick parylene C layer deposited on a 4-inch Si carrier wafer. A 10nm thick layer of chromium and 250 nm layer of gold was then deposited by electron beam evaporation to form the photolithographically defined metal trace connections for the contacts. The Pt-based contact materials were deposited using a direct current (DC) sputtering system. Then a conformal 3.5 μm thick parylene C top passivation was formed using vapor deposition. The PEDOT:PSS was spun-cast on to the electrode and patterned using a sacrificial parylene C layer (Ganji et al., 2017).

**FIGURE 2:**
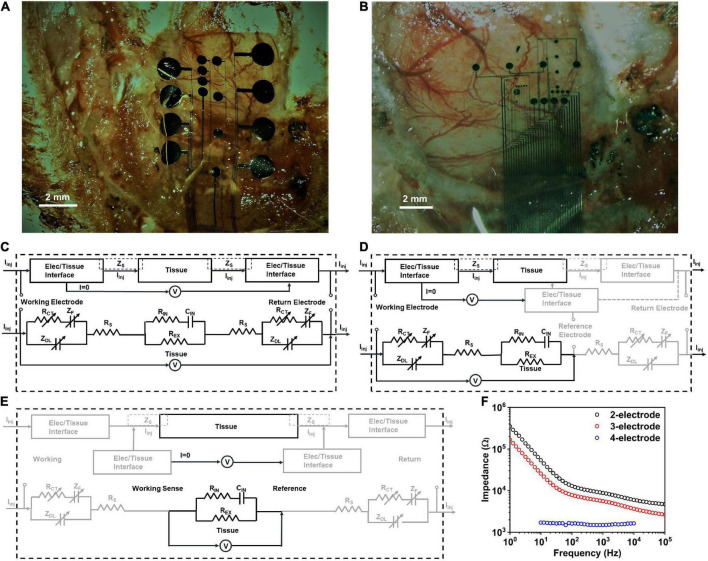
Electrode positioning *in vivo* for **(A)** small contact and **(B)** large contact arrays. The observed impedance elements in **(C)** 2-contact, **(D)** 3-contact and **(E)** 4-contact electrochemical impedance spectroscopy (EIS) measurements, *in vivo*. **(F)** Electrochemical impedance spectra *in vivo* for a PtNR contact with a diameter of 200μm in the 2-, 3-, and 4-contact configurations.

Four electrode designs were used for the parametrization of the electrode contacts, two for *in vivo* (Figures 2A,B), and two for benchtop experiments. The small electrode design encompassed contact diameters of 200 μm, 100 μm, 50 μm and 30 μm. The large electrode design encompassed contact diameters of 1000 μm, 600 μm and 400 μm. The *in vivo* electrode design included openings adjacent to the contact in the parylene C film to allow the perfusion of the cerebrospinal fluid and to attain a better adhesion to the cortical surface. To study the effect of current spreading and the geometric correction factor of the electrode design on the electrochemical performance, five inter-contact edge-to-edge separations (1.5*D*, 2*D*, 3*D*, 4*D*, and 5*D*) were studied where *D* is the contact diameter. The 400 μm, 600 μm, and 1000 μm larger diameter PtNR electrodes were designed with one hundred 40 μm, 60 μm, and 100 μm diameter contact openings within each contact to make the total exposed area of the electrode equivalent to that of a circular contact with the designed contact diameter.

Benchtop testing was conducted using a Sigma-Aldrich phosphate buffered solution (PBS) consisting of 0.022M Na_2_HPO_4_ (pH = 7.2 ± 0.2). The 4-contact EIS measurements were performed by under varying concentrations of NaCl dissolved in de-ionized water, and the resulting conductivity was measured using an Extech conductivity meter. The acute recordings were performed on 6 male Sprague-Dawley rats over the course of 5 acute experiments, each experiment lasting around 4 h. All experiments were performed under the guidelines stated in the University of California San Diego Institutional Animal Care and Use Committee (IACUC) protocol S16020. The animals were anesthetized prior to surgery using Isoflurane and Ketamine, and constant anesthesia was maintained throughout the course of the experiment using Ketamine. At the end of the experiment, the animal was euthanized with a lethal injection of Sodium Pentobarbital.

To investigate the nature of the electrochemical contact of the electrode with the saline or *in vivo* tissue, we performed electrochemical impedance spectroscopy measurements in the 2, 3 and 4-contact setup, as shown in Figure 2F (Merrill, 2021; Vatsyayan et al., 2021). The 4-contact impedance measurement setup, shown in Figure 2E, involves using 4 contacts on the electrode to measure the impedance contribution of the surrounding media (Robillard and Poussart, 1977; Zimney et al., 2007). The 4 contacts were spaced equally, with the two outer contacts being used to inject current into the media, and the two inner electrodes used to passively monitor the voltage drop in the media. Using this technique, we eliminate the effect of the electrode-media interface on the impedance measurement which for a known injected current measures only the bulk media impedance (Hargreaves and Millard, 1962).

We then investigated the 3-contact impedance of the electrode, with the working contact on the electrode used to inject the current, and the reference contact used to passively set the potential to the equilibrium potential of the media and the counter electrode used for the current return from the media, completing the circuit, as shown in Figure 2D. We subtract the 4-contact impedance from the measured 3-contact impedance measurement, which allows us to delineate the impedance of the electrochemical interface at the working electrode. Finally, we measured the electrochemical impedance in the 2-contact setup (Figure 2C), with two contacts on the electrode, one working and one acting as both the counter and reference electrode. This is the most used setup for clinical stimulation and is the configuration we will focus on in this investigation. Setting one contact as both the reference and counter means that the impedance measured will now be comprised of the electrode-media interface impedance at both the working contact and the counter electrode contact.

**FIGURE 3:**
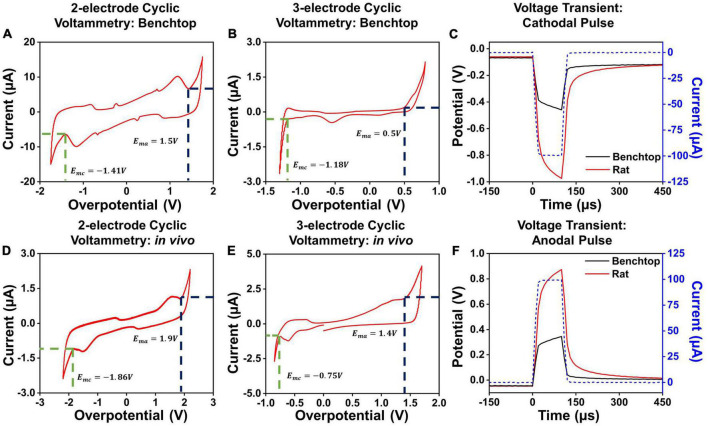
Cyclic voltammetry measurements in a 2-contact configuration in **(A)** a benchtop and **(D)** *in vivo* setup. Cyclic voltammetry measurements in a 3-contact configuration in **(B)** a benchtop and **(E)** *in vivo* setup. Example voltage transients for a 100 μA, 100 μs **(C)** cathodal and **(F)** anodal pulse *in vivo* and in benchtop settings across a PtNR contact with a diameter of 200 μm.

To determine the hydrolysis window in the two-contact setup, we performed cyclic voltammetry measurements for each contact diameter and separation. The cyclic voltammetry measurements were performed by gradually increasing the applied potential at the electrode, at 200mV/s, and sampling the resultant current flow (Evans et al., 1983; Kissinger and Heineman, 1983). For low potentials applied on the electrode surface, the charge injection occurs due to capacitive current and through reversible electrochemical reactions. As higher potentials are applied to the electrode, irreversible Faradaic reactions at the electrode-media interface begin to dominate. This is marked by a sudden unrestricted increase in the measured current as the electrode interface begins to either oxidize or reduce (Van Benschoten et al., 1983; Daubinger et al., 2014; Ismail et al., 2019). The point at which we observed a sudden, unrestricted increase in current was considered as the electrochemical safety limit for the electrode which is the maximum potential that can be built up on the electrode surface during the charge injection process (Rand and Woods, 1972; Doña Rodríguez et al., 2000). The electrochemical potential at which these irreversible reactions begin to dominate is considered the electrolysis window, commonly referred to as the water window for the case of hydrolysis (Brummer and Turner, 1977). We establish the cathodal, *E*_*mc*_, and anodal, *E*_*ma*_, safety thresholds from these plots, and subsequently use these to determine the current safety thresholds. Examples of such cyclic voltammetry measurements and extractions for PtNR contacts with a diameter of 200μm in the 2- and 3-contact configurations for benchtop and *in vivo* experiments are shown in Figures 3A,B,D,E. We observe that the water electrolysis window widens in the two-electrode setup, as compared to the three-electrode setup, as the potential drop in the two-electrode configuration is measured across both the working and the counter electrodes. This voltage drop is asymmetric, since the charge injection mechanism varies between the injecting (working) and extracting (counter) contact, and consequentially the observed current flow corresponding to each mechanism is different. Further, the electrolysis limit depends on the nature of the media surrounding the tissue, and we observe that the electrolysis window is wider *in vivo* compared to benchtop.

Here, we will focus primarily on cathodal-first stimulation, since typical experimental paradigms involve biphasic stimulation pulses with a cathodal first charge injection phase (Cogan et al., 2006; Bronzino and Peterson, 2020). We established the maximum charge injection capacity and the stimulation current threshold for each electrode by measuring the voltage transients (Figures 3C,F). We employed a current clamped stimulation, in which a square wave pulse is applied to the electrode, where the excitation voltage amplitude was set to achieve the desired current level. The potentials observed in the voltage transient measurements are comprised of the drop at both the contacts and the media. The current value for which the electrode potential at the interface equals the safety limits determined from the CV measurements is considered the current injection limit (*I*_*max*_) for the electrode. The variations in the observed cathodal excitation were studied as a function of the stimulation setup (current amplitude and pulse width) as well as the electrode design parameters (contact material, size and separation), and the resulting trends were fit into a predictive equation for each electrode design parameter. All the measured fits and analyses presented in this manuscript were performed using non-linear least squares regression to obtain the best fit for the measured data. The fits were optimized by maximizing the adjusted *R*^2^ value of the observed and modeled data (Johnson and Frasier, 1985; Ostertagová, 2012).

## Results

### Electrochemical impedance spectra

**FIGURE 4:**
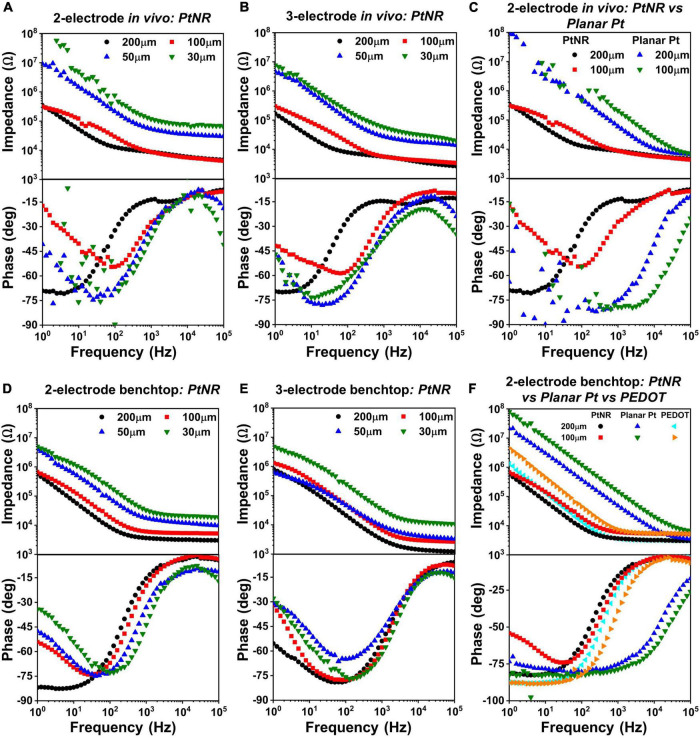
Diameter-dependent measured EIS spectra *in vivo* for PtNR contacts in **(A)** 2-contact configuration and **(B)** 3-contact configuration, and in benchtop for **(D)** 2-contact configuration and **(E)** 3-contact configuration. Side-by-side comparison of 2-contact PtNR and planar Pt contacts **(C)** *in vivo*. **(F)** Side-by-side comparison in benchtop, of PtNR, planar Pt and PEDOT.

The EIS for different electrode materials, contact sizes and contact geometry were measured *in vivo* (Figures 4A–C) and in a benchtop saline setup (Figures 4D–F). The measured results indicate that the PtNR electrodes have a consistently lower impedance than the PEDOT and planar Pt electrode, due to the increased geometric surface area of the contact and its increased electrochemical activity (Ganji et al., 2019).

The magnitude and phase of the electrochemical impedance of the electrode plays a critical role in determining the current injection capacity and safety limits for a stimulating electrode: a contact with a small geometric surface area and small double-layer capacitance, e.g., with a higher reactance will observe higher potential excursions for a given stimulation current compared to contact with a higher geometric surface area, higher double-layer capacitance and lower reactance. Given that a higher excursion potential will be built over the contact with a smaller geometric surface area, lower currents injected in these contacts can induce water hydrolysis compared to contacts with larger geometric surface area deeming the latter safer for stimulation.

At higher frequencies, the impedance spectra are dominated by the resistive elements of the circuit, specifically the spreading resistance and the bulk impedance. At lower frequencies, the capacitive elements of the circuit, in particular the double layer capacitance, begin to dominate, and the phase of the EIS spectra reaches nearly –90° in saline. At very low frequencies, faradaic reactions begin to dominate, and charge injection occurs due to the movement of ionic species across the interface. The resultant species have time to diffuse away from the electrode surface, and the charge injection process is no longer limited by the presence of ionic species in the vicinity of the contact. This effect is observed in the measured phase spectra where the phase begins deviating towards 0°.

Figures 4A,D show the variation of the EIS spectra of PtNR electrodes in the 2-contact configuration *in vivo* and in benchtop, respectively. The observed impedance increases with decreasing contact diameter indicating that the spreading resistance and the double layer capacitance decrease with decreasing diameter. In benchtop measurements for the larger contacts, the minima of the phase of the EIS spectra is closer to –90°, which is indicative of the effectiveness of the double-layer capacitance, *C*_*DL*_, and the minimal role of the charge transfer resistance, *R*_*CT*_, in charge injection. This effect is less pronounced *in vivo* due to a lower double layer capacitance (and a correspondingly higher impedance), which lowers the effectiveness of the double layer charge injection process and consequently manifests as a lower charge injection limit. The benchtop spectra indicate that the contribution of the charge transfer resistance at 1Hz becomes more significant for smaller contacts, which have lower *C*_*DL*_ and higher *R*_*CT*_. This trend is also consistently observed in the 3-contact configuration, both *in vivo* and in benchtop. Further, we also find that the impedance spectra is higher *in vivo* than in benchtop. This consequentially leads to lower current injection thresholds *in vivo*, in line with previously observed results (Black et al., 2018). The variation of each circuit element for the PtNR electrodes as a function of the contact geometry is shown in Supplementary Figure 1, and the corresponding numerical values are shown in Supplementary Table 1.

Figures 4C,F compare the EIS spectra for the PtNR and the planar Pt contacts *in vivo* and in benchtop. Since PtNR has 3-dimensional topography (e.g., rods) at its interface, its geometric surface area is significantly higher than that of the 2-dimensional planar Pt contact material. The corner frequency at which the series resistance elements in the media begin to dominate is also significantly higher for both smaller contacts, as well as for planar Pt contacts when compared to PtNR contacts. This indicates that the resistive and capacitive elements scale with contact sizes differently when the contact material is changed. For the planar Pt contact shown in Figure 4C, the phase and impedance values below 100Hz show non-idealities as the observed impedance of the contact approaches the measurement limit of the instrument. We observe that for the low-impedance, high surface area PtNR and PEDOT contacts, the impedance spectra are identical at higher frequencies. This indicates that the high frequency impedance is dominated by the impact of current crowding due to volumetric conduction in the vicinity of the contact and is independent of the contact material. The impedance of the planar Pt contact also tends toward this value at very high frequencies (close to 100 kHz). The corner frequency for both PtNR and PEDOT electrodes is significantly lower than that of planar Pt, which is indicative of their superior stimulation capabilities.

**FIGURE 5:**
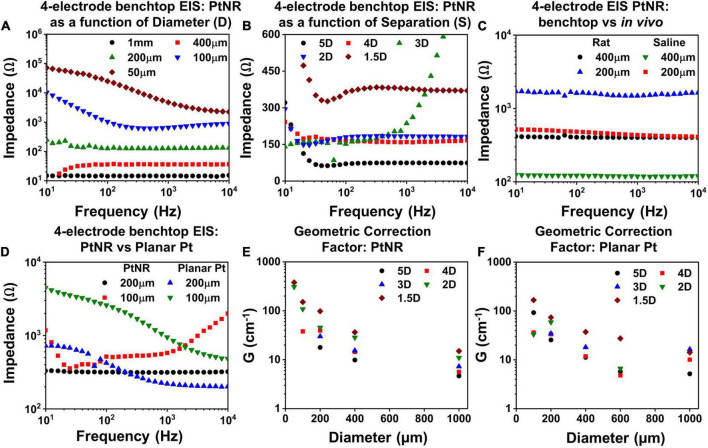
Measured 4-contact EIS spectra for PtNR contacts as a function of **(A)** diameter for inter-contact separation of 1.5D in benchtop, **(B)** inter-contact separation for a contact diameter of 200μm and **(C)** measurement media: *in vivo* versus benchtop for two diameters with inter-contact separation of 1.5D. **(D)** Comparison of the 4-contact EIS Spectra for PtNR and planar Pt contacts with diameters of 100 μm and 200 μm. Geometric correction factor as a function of diameter for different inter-contact separation for **(E)** PtNR contacts and for **(F)** planar Pt contacts.

Finally, to delineate each element of the electrode’s interfacial impedance, we measured the EIS in a 4-contact configuration (Figure 5A). The 4-contact configuration of the electrode allowed us to extract the impedance of the surrounding media, which did not form a part of the interface and hence does not directly impact the electrochemical safety of stimulation. The observed voltage drop in the surrounding tissue depends on the current spreading around the contact and will hence vary as a function of the separation between the working contact and the counter contact.

To characterize the effect of current spreading, we measured the 4-contact EIS spectra for contact separations of 1.5*D*, 2*D*, 3*D*, 4*D*, and 5*D* (where *D* is the diameter of the contact). We observe from Figure 5B that the 4-contact impedance increases as the inter-contact separation reduces. This arises due to the increase in the current crowding near the contact when the inter-contact separation is small and the volumetric conduction around the contact is limited. The measured 4-contact impedance is smaller in benchtop compared to *in vivo*, as seen in Figure 5C, which is consistent with the higher reported values of tissue impedance (Stoy et al., 1982; Latikka et al., 2001). The dependence of the 4-contact impedance on the material of the injecting contact is negligible as expected and seen in Figure 5D. The 4-contact impedance depends primarily on the geometry of the injecting and the counter contact. We also measured the 2-contact EIS as a function of contact separation (Supplementary Figure 2) and quantified the effects of contact separation by calculating the geometric correction factor of the contact.

### Geometric correction factor

The geometric correction factor, G, quantifies the contribution of the media in the observed EIS spectra. Previous studies have shown that G is a function of the inter-contact separation (Rymaszewski, 1969). For a solution of resistivity ρ, and for a measured resistance of *R*, *G* can be expressed as:


(1) $$G=\frac{4\,\pi \,R}{\rho }$$

The term G/4π can be calculated from the inverse of the slope of a measured ρ-*R* plot. To calculate *G*, we used NaCl solutions with concentrations ranging from (0.1 g/100 ml to 1 g/100 ml). Figures 5E,F show the geometric correction factor for the PtNR and planar Pt contacts. We observed that *G* tends to decrease as the inter-contact separation increases. This trend can be explained by the impact of non-uniform current spread between the contacts. When the contacts are placed close to each other, the current spreading occurs in a limited volume of the media surrounding the contact, and the observed impedance is therefore higher. As the inter-contact separation increases, the injected current spreads over a larger volume, and the overall impedance becomes lower and consequently *G* decreases compared to that for smaller inter-contact separation.

This trend was also observed as we decreased the contact size of the injecting electrode. For smaller contacts, the current spreads over a smaller volume in the surrounding media, which leads to a higher observed resistance (Figure 5E). The role of the contact material itself is less significant in determining *G*, since this measurement eliminates the effect of the current injecting interface itself and accounts for geometric effects of current injection in the tissue in the vicinity to the contact.

### Determining current safety limits (i_*max*_)

**FIGURE 6:**
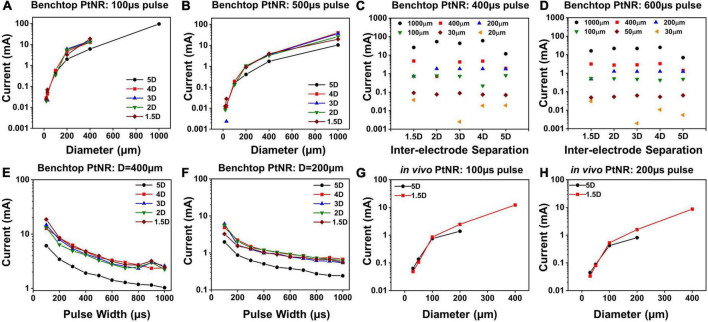
Maximum injectable current for PtNR contacts as a function of **(A)** diameter for a 100 μs pulse and for different inter-contact separation in benchtop setting, **(B)** diameter for a 500 μs pulse and for different inter-contact separation in benchtop setting, **(C)** inter-contact separation for a 400 μs pulse and for different diameters in benchtop setting, **(D)** inter-contact separation for a 600 μs pulse and for different diameters in benchtop setting, **(E)** injected pulse width for a 400 μm contact diameter and for different inter-contact separation in benchtop setting, **(F)** injected pulse width for a 200 μm contact diameter contact in benchtop setting, **(G)** diameter for a 100 μs pulse for two different inter-contact separations *in vivo*, **(H)** diameter for a 200 μs pulse for two different inter-contact separations *in vivo*.

Figure 6 shows the current injection limits for each electrode and contact configuration. We observe that *I*_*max*_ increases as the contact diameter increases, as seen in Figures 6A,B. This trend is expected since larger contacts have higher *C*_*DL*_ and more active surface area for charge injection and can hence allow injection of higher currents. However, we observed that the increase in *I*_*max*_ is non-linear, and for larger contacts, the charge injection density reduces. For bipolar stimulation, current is injected between two contacts and for larger contacts, the field lines and consequently current vector fields are focused on the opposing perimeters of the contacts, meaning that a smaller fraction of the surface area is effective in current injection. Due to the limited area at the edge of the contact for charge injection, a smaller total current may be injected than would have been predicted from currents injected in smaller contacts with a uniform surface area (charge density) assumption (Figures 6A,B). For a fixed diameter above 200μm, a larger inter-contact separation amplifies this effect and as a result, the total safe current decreases (Figures 6C,D).

The dependence of *I*_*max*_ on the inter-contact separation is shown in Figures 6C,D. For the larger electrode contacts, we observe that as a general trend, *I*_*max*_ is lower for higher inter-contact separation (5D). For larger inter-contact separation, the effect of non-linearities due to the fringing fields at the edge of the contact becomes more pronounced, and the charge injection through the electric double layer is less effective. For smaller contact separations, the fringing fields are less pronounced, and the electric field is more uniform across the whole contact area which improves the charge injection through the electric double layer at the contact. As we increase the pulse width of the injected current, *I*_*max*_ decreases, since we inject more charge into the medium at the interface per phase while simultaneously providing a longer time for ionic species to migrate away from the interface. This drop is non-linear because the electrochemical interface itself changes with the built-up potential across it. With higher interface potentials, Faradaic current processes start to dominate the charge transfer at the electrode-tissue interface. Effectively, the Faradaic branch impedance drops and hence the overall impedance drops. As can be seen from the voltage transients in Figures 3C,F, this leads to a non-linear charging of the interface (as opposed to a linear charging expected from a purely capacitive interface). Therefore, for longer pulse widths, we can inject more charge, which explains the non-linear decrease in *I*_*max*_ with pulse width.

Finally, we calculated the current safety limits *in vivo* and compared it to the benchtop limits. It has been shown previously in literature that the electrochemical impedance is higher *in vivo*, which indicates that we expect to see lower current thresholds. This is consistent with our observations of higher impedances *in vivo* (Figures 4A,D), and correspondingly lower values of *I*_*max*_, as shown in Figures 6G,H.

## Parameters affecting the observed potential at the electrode (*V*_*elec*_)

To predict the variation of the electrode potential, we study the impact of the design parameters, including the injected current (*I*_*inj*_), the pulse width of biphasic symmetric stimulation (*t*_*pw*_) the choice of the electrode material and the contact diameter. These are important choices that researchers and physicians typically face when designing a new stimulation paradigm, and it is crucial to understand the role of each parameter in determining the safety threshold of stimulation.

### Injected current

**FIGURE 7:**
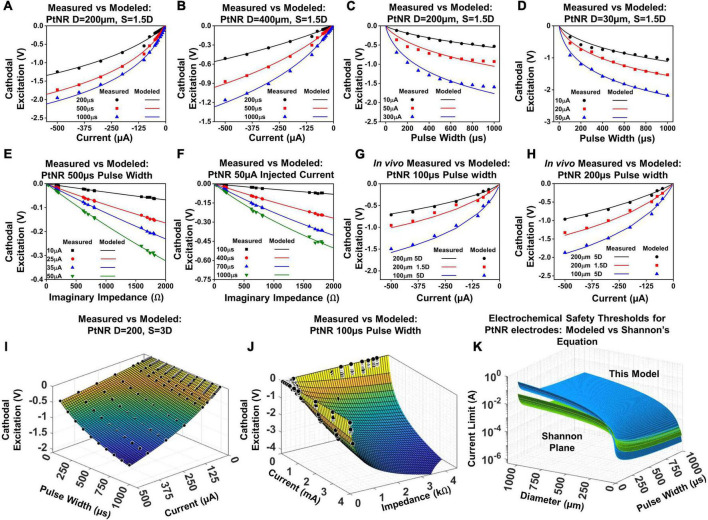
Modeled vs. predicted cathodal excitation in benchtop as a function of **(A,B)** current for a **(A)** 200 μm and **(B)** 400 μm contact, **(C,D)** pulse width for a **(C)** 200 μm and **(D)** 400 μm contact, **(E,F)** Reactance at 10 kHz for a **(E)** 200 μm and **(F)** 400 μm contact. **(G)** and **(H)** *In vivo* comparison of the equation performance for measured cathodal excitation in a rat experiment. **(I)** Fit for the cathodal excitation as a function of the pulse width and current. **(J)** Fit for the cathodal excitation as a function of the impedance and current. **(K)** The safety limit determined by the modeled equation in this work in comparison to the limits defined in the Shannon’s equation.

The injected current (*I*_*inj*_) pre-dominantly determines the potential excitation at the electrode contact (*V*_*elec*_). The amount of charge injected through the interface directly impacts the amount of potential built across the interface. However, the relation between *I*_*inj*_ and *V*_*elec*_ is non-linear, as observed from Figures 7A,B. This follows from our previous discussion regarding the decay of the observed electrochemical impedance with interfacial potential. Specifically, we can model *V*_*elec*_ as a function of *I*_*inj*_ as follows:


(2) $$V_{e\,l\,e\,c}=-l\,n\,\left(k_{1}\,{\left|I_{i\,n\,j}\right|}^{k_{2}}+1\right)$$

The variables *k*_1_ and *k*_2_ model the variation of the electrode excitation due to other external stimulation parameters, including the electrochemical impedance of the stimulating contact, the geometric correction factor, the pulse width of the injected current and the contact material itself. Figures 7A,B showcase the fit of this equation against measured data in a benchtop setup.

### Pulse width of injected current

The pulse width (*t*_*pw*_) of the injected current is an important design parameter for stimulation experiments, and it is common for experimental paradigms to incorporate longer, or shorter pulse widths based on the specific application (Baldwin et al., 2006; Yearwood et al., 2010; Lee et al., 2011). Figures 7C,D show the variation of the cathodal excitation potential as a function of the input pulse width. The variation *V*_*elec*_ with pulse width follows similar trends as the variation of the injected current since the total potential build up depends on the net value of injected charge. However, the voltage build up is non-linear, since the impedance is a function of both the built-up potential and frequency, both of which vary with the duration of the injected pulse. The observed *V*_*elec*_ is therefore expressed as:


(3) $$V_{e\,l\,e\,c}=-l\,n\,\left(k_{3}\,t_{p\,w}^{k_{4}}+1\right)$$

The variables *k*_3_ and *k*_4_ capture the variation of the cathodal excitation as a function of the other stimulation and design parameters. Figures 7C,D show the fit of Equation (3) with the observed experimental data.

### Electrochemical interface impedance

We next investigate the nature of the electrochemical interface. The charge stimulation threshold is determined by the build-up of charge across the double layer capacitance whose reactance plays a major role in determining *V*_*elec*_. Figures 7E,F show the dependence of *V*_*elec*_ on the reactive impedance. The magnitude of the imaginary impedance (reactance) is taken at a frequency of 10kHz. The duration of the injected pulse varies from 100–1000 μs, which corresponds to a principal frequency of 10–1 kHz. Therefore, to capture the effect of the electrochemical impedance on the cathodal excitation, we invoked the impedance in the frequency range corresponding to the principal frequency of the injected pulse (noting that square waves also contain harmonics which are neglected). The electrochemical impedance restricts the amount of charge delivered through the interface, and hence couples to the built-up potential at the electrodes as:


(4) $$V_{e\,l\,e\,c}=-l\,n\,\left(k_{5}\,{\left|Z_{i\,m\,a\,g}\right|}^{k_{6}}+1\right)$$

*V*_*elec*_ as a function of the imaginary impedance is logarithmic; for larger contacts, the proportion of active sites is lower, which introduces non-linearities in the measured *V*_*elec*_ as a function of impedance, and for smaller contacts, the full contribution of all active sites is mandated, resulting in a weaker slope and in essence a higher charge injection capacity.

By superposition of Eqs. (2–4), we can now express *V*_*elec*_ as:


(5) $$V_{e\,l\,e\,c}=a\,\left[l\,n\,\left(b\,{\left|I_{i\,n\,j}\right|}^{k_{2}}\,t_{p\,w}^{k_{4}}\,{\left|Z_{i\,m\,a\,g}\right|}^{k_{6}}+1\right)\right]$$

where *a* and *b* are process dependent parameters which account for the dependence of *V*_*elec*_ on the electrode design, the experimental setup, the electrode material, the interface with the surrounding media and general variability in the injection process. The parameters *k*_2_ and *k*_4_ are shown in Supplementary Table 2 for PtNR, planar Pt and PEDOT.

When *V*_*elec*_ in Equation (5) reaches *E*_*mc*_ for any given *I*_*inj*_, *t*_*pw*_, or *Z*_*imag*_, *I*_*inj*_ becomes *I*_*limit*_, which is the highest current that can be injected into tissue before electrolysis occurs. Alternatively, we can express *I*_*limit*_ by setting *V*_*elec*_ = *E_*mc*_*, as follows:


(6) $$\left|I_{l\,i\,m\,i\,t}\right|={\left[\frac{1}{b\,t_{p\,w}^{k_{4}}\,{\left|Z_{i\,m\,a\,g}\right|}^{k_{6}}}\,\left(e^{\frac{E_{m\,c}}{a}}-1\right)\right]}^{\frac{1}{k_{2}}}$$

Equation (6) illustrates the exponential dependence of the injected current limit on the built-up potential at the electrode-media interface. The exponential dependence of the charge injection current on the built-up potential or overpotential is consistent with known expressions for the charge injection process (Merrill, 2021), used in a different context than the electrochemical safety limits developed here.

## Discussions

### Validation of model results

The parameters listed above with their full dependences on diameter and pulse width for Equations (2) – (5) were first calibrated on the benchtop data sets. To validate the model *in vivo*, we performed the voltage transient measurements for a subset of PtNR and planar Pt contacts on the rat brain. The electrode potential was calculated for pulse widths of 100 μs and 200 μs, for contact diameters of 30 μm, 50 μm, 100 μm and 200 μm, and inter-contact separations of 1.5D and 5D. Figure 7 shows the comparison between the modeled and measured results, first by varying individual parameters of the model, and then comparing the measured cathodal excitation *in vivo* against the predicted cathodal excitation based on the *in vivo* EIS of the electrode (Figures 7G,H). We observed a reasonably good agreement between the predicted and measured cathodal excitation voltage.

Equation (5) models the variation of the cathodal excitation as a function of 3 independent parameters, *I_*inj*_, t_*pw*_* and *Z*_*imag*_. To investigate the performance of the model more extensively, we compared the measured and predicted results by simultaneously varying I_*inj*_ and *t*_*pw*_ (Figure 7I), and *I*_*inj*_ and *Z*_*imag*_ (Figure 7J). We observed good agreement between the measured and predicted values by our model with an observed *R*^2^ of 0.997 and 0.991, respectively.

For both benchtop and *in vivo*, we observed a variance between the measured and the modeled exponent for the *t*_*pw*_ as the amount of charge injected shows a non-linear behavior with the injected pulse width. Our results show that the exponential parameter *k*_4_ in Equation (5) tends to be close to 0.9 for PtNR, although it can deviate between 0.7 and 0.9 based on the electrode geometry. This indicated the presence of significant non-linearities in the charge injection process, arising due to the variation of the behavior of charge injection sites on the electrode with time and built-up potential. We found that *k*_4_ can vary significantly with the contact material. *k*_4_ was ∼ 0.8 for PEDOT which is lower than that for PtNR and *k*_4_ was ∼ 1.1 for planar Pt which higher than that of PtNR (Supplementary Table 2). However, independent of the choice of material for stimulation, we observe that the model fits well with the observed experimental data, as seen in Supplementary Figure 3, with an adjusted *R*^2^ value of 0.997, 0.98 and 0.996 for the fit of the model on a 200μm contact for PtNR, planar Pt and PEDOT.

### Electrochemical safety limit with our model versus Shannon’s limit

With knowledge of the individual parameters affecting the performance of the electrode contact, we were able to evaluate the predicted safety limit for the electrode and compare it to the previously established tissue safety thresholds from Shannon’s limits. PtNR has a very high effective geometric surface area, and as such, we observe very low electrochemical impedances at very small sizes as well. This allows us to inject significantly higher currents than conventional electrode materials, and the electrochemical safety limit for the electrode contact is consequently significantly higher *in vivo* than that predicted by the Shannon’s equation, as shown in Figure 7K. Since the Shannon’s equation relates the injected charge density with the injected charge, we parametrize the observed 1kHz impedance of the electrodes as a function of contact diameter. We observe that *Z*_*imag*_ decays non-linearly with the diameter, and the observed dependency was modeled as:


(7) $$Z_{i\,m\,a\,g}=\alpha \,D^{d_{1}}$$

where the parameter α is an intrinsic property of the contact material, and *d*_1_ models the exponent for decay of the electrode impedance with increasing contact size and is equal to –1.67 for PtNR and –1.61 for planar Pt. However, planar Pt has a lower effective surface area for charge injection, and thereby has a higher electrochemical impedance (Figures 4C,F). Therefore, the choice of the electrode material will play a critical role in determining the safety threshold for stimulation, which is a property that is not captured in either the Shannon’s equation or the routinely used 30μC/cm^2^ and 4nC/ph safety thresholds (McCreery et al., 1990, 2002).

The Shannon’s equation is typically written as:


(8) l⁢o⁢g⁢D=k-l⁢o⁢g⁢Q

where *D* is the charge density per phase of injected charge, represented in μC/cm^2^/ph, and *Q* is the total charge injected per phase, represented in μC/ph. *k* is an empirically determined parameter for setting the tissue damage threshold, usually considered to be equal to 1.8. The total charge injected, *Q*, is equal to the injected current times the pulse width, and *D = Q/A*, which allows us to re-write Equation (8) as:


(9) $$l\,o\,g\,\left(\frac{I_{i\,n\,j}\times t_{p\,w}}{A}\right)=k-l\,o\,g\,\left(I_{i\,n\,j}\times t_{p\,w}\right)$$

where *A* is the area of the injecting contact. For circular contacts of diameter *D*, we can re-write Equation (9) as:


(10) $$I_{l\,i\,m\,i\,t_{S\,h\,a\,n\,n\,o\,n}}=\frac{D\,\sqrt{\pi \,10^{k}}}{2\,t_{p\,w}}$$

The Shannon’s equation predicts a linear dependence of the injecting current on the contact diameter and the pulse width. However, we observed in our experiments that the dependence of the current safety limit is non-linear. By substituting Equation (7) into Equation (6), we obtain:


(11) $$\left|I_{l\,i\,m\,i\,t,t\,h\,i\,s\,w\,o\,r\,k}\right|={\left[\frac{\alpha \,D^{-d_{1}\,k_{6}}}{b\,{\left(t_{p\,w}\right)}^{k_{4}}}\,\left(e^{\frac{E_{m\,c}}{a}}-1\right)\right]}^{\frac{1}{k_{2}}}$$

The parameters *α, a, b, k_2_, k_4_* and *k*_6_ model the non-linear dependence of the current safety limit on the experimental design parameters, especially as we migrate from the large diameter macroelectrodes to small diameter microelectrodes. Supplementary Figure 4 shows the effect of changes in the electrochemical interface (contact material and surrounding media) captured by our model that is not accounted for in the Shannon’s equation. Typically for microelectrodes, we observe that the impedance decay is a non-linear function of the contact size (D), which correspondingly leads to a non-linear increase in the maximum current that can be safely injected, as discussed before. The choice of a suitable *k* from the Shannon’s equation is done purely empirically, whereas the electrochemical safety for charge injection will depend on the electrolysis limit for the contact (*E*_*mc*_), which in turn will be material and media dependent. Finally, the bias dependent non-linearities at the interface of the electrode indicate that the variation of the safety limit will depend not on the absolute value of the charge injected into tissue *(Q_*inj*_)*, but rather on the setup used to inject charge into tissue (i.e., the pulse width and the amplitude of the injected pulse). Therefore, it becomes crucial to accurately capture the effects of each charge injection parameter in the current injection process, which is what this model aims to achieve.

### Model validation on held-out test data

**FIGURE 8:**
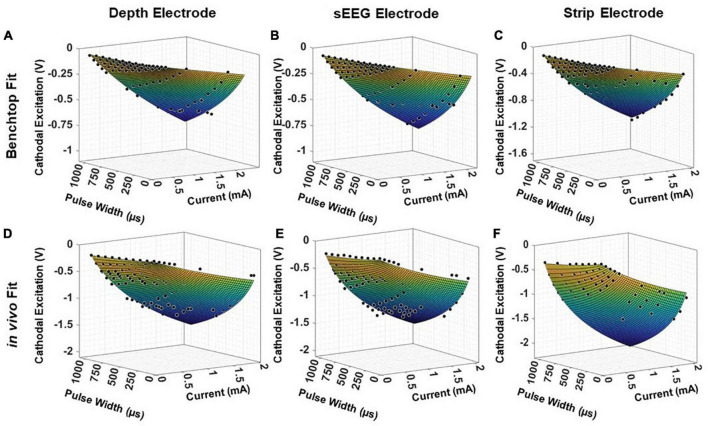
Fitting results for clinical electrodes measured on a pig’s cortex, plotted as a function of the magnitude of the input current and the pulse width, for a **(A,D)** depth electrode, **(B,E)** sEEG electrode, and **(C,F)** strip electrode, in benchtop and *in vivo* measurements, respectively.

To validate the universal applicability of the model, we tested the model predictions on previous electrochemical characterization carried out in an acute pig experiment with clinical depth, sEEG and strip electrodes. This is a held-out test set for the model, with the electrode used in the experiment not used in any modeling and optimization. We first analyze the performance of the test electrodes in a benchtop setup, performing EIS and voltage transient measurements and fitting the resultant data into the model presented in Equation (5) (Figures 8A–C). This serves as the baseline, allowing us to extract the parameters *a, b, k_2_* and *k*_4_. The EIS spectra is used to obtain the imaginary impedance of the electrode at 10kHz. To determine the safety thresholds *in vivo*, we measure the EIS post-implantation to extract the imaginary impedance at 10 kHz. The parameters *a, b, k_2_* and *k*_4_ are intrinsic to the electrode, and hence can be expected to remain the same. For the 2 insertion-type electrodes (sEEG and depth), the most optimal value of *k*_6_ was 1.08, whereas for the surface electrode (strip), the optimal value of *k*_6_ was closer to 1.04. The resultant fit for each electrode versus the experimentally measured *in vivo* data is plotted in Figures 8D–F. We observe good agreement between the modeled and fit data (*R^2^> 0.99)*. We also ran a paired *t*-test on each fit, with the null hypothesis that the measured and predicted cathodal excitations arise from the same dataset, and the results indicated no statistically significant difference between them, with a *p > 0.9*.

## Limitations

The experiments were formulated to study the impact of design parameters on the electrochemical performance of micro-contacts. However, the electrochemical charge injection process is inherently non-linear, and there are technical limitations to this approach. Measurements were made across a large range of currents and pulse widths for square waves, but more studies can be performed for different stimulus waveforms, and the choice of the waveform will affect the electrochemical safety threshold. Typical clinical stimulation can be chronic (e.g., DBS for epilepsy and Parkinson’s Disease) or acute (stimulation mapping during neurosurgery). However, our experimental results only measure the performance of the electrode in a short-term (less than 5 h) acute study. While we repeated our benchtop measurements for different devices with the same electrode material and geometry, the *in vivo* measurements were not repeated across multiple samples and tested for longer implant durations.

We use the electrochemical impedance of the electrode contact as an estimate of the potential performance of the contact. However, variations in the fabrication process can lead to non-linearities in the electrochemical performance of the electrode contact. For micro-contacts, point defects in the contact are no longer averaged over a large area on the contact, which can lead to variability between samples. Further, despite varying a wide range of process parameters, we still cannot create a big enough data set to account for all possible variations in the charge injection process. The electrochemical interface consists of both capacitive and faradaic elements, and the charge injection mechanism switches between the two depending on the frequency and amplitude of the injected pulse. The exact nature of the interface is highly non-linear and complex, and changes with almost all parameters of the electrode and experimental design. Due to multiple changing parameters, we do not evaluate the electrode performance as a function of the individual elements of the interface, to avoid overfitting of data from a comprehensive, yet relatively limited dataset.

While electrolysis is generally considered a leading cause of tissue damage as well as electrode failure, it is not by any means the only mechanism of damage, and we did not perform histological evaluations of the stimulated tissue to investigate other damage mechanisms. Chronic, repeated stimulation below the electrolysis window has been known to cause significant neurobiological changes in nearby neurons and can affect the neural networks they form a part of. The precise nature of these changes and the resulting harm can vary case to case. These electrochemical safety limits studied here should only be considered a tentative upper bound for stimulation of the electrodes studied.

## Conclusion

We investigated the stimulation performance of thin film flexible surface electrodes with PtNR, planar Pt and PEDOT as the stimulating contact and established the electrochemical stimulation safety windows. The charge injection capacity of the electrodes increases non-linearly with pulse width and contact size because of the non-linearities in the interfacial elements of the electrode-tissue and electrode-saline interface. Further, fundamental digressions in the nature of the electrochemical interface of the electrode necessitates the characterization of the interface *in vivo*, and benchtop saline measurements aren’t sufficient to determine safety limits. We established a procedure to characterize and extract the functional dependence of the cathodal excitation as a function of the experimental design parameters, i.e., the electrode material and contact size, the injected current and the duration of the injected pulse and developed a model that accurately predicted these dependencies both *in vitro* and *in vivo*. We validated our model against *in vivo* measurements for both thin film and clinical electrodes and saw reasonably good agreement with our measurements. We propose that with characterizations illustrated in this work that the electrochemical safety limits can be predicted for any electrode contact material or stimulation paradigm.

## Data availability statement

The original contributions presented in this study are included in the article/Supplementary material, further inquiries can be directed to the corresponding author.

## Ethics statement

The animal study was reviewed and approved by UC San Diego IACUC, iacuc@ucsd.edu, protocol S16020.

## Author contributions

SD conceived and led the work. RV carried out all experiments and led the data analysis under SD’s supervision. RV and SD wrote the manuscript. Both authors contributed to the article and approved the submitted version.
