# Supplementary material for: A universal model of electrochemical safety limits in vivo for electrophysiological stimulation
Source: Front Neurosci. 2022 Oct 6;16:972252. doi: 10.3389/fnins.2022.972252 (PMC9582612; doi:10.3389/fnins.2022.972252)
Supplement: Supplementary file 1 [file Data_Sheet_1.docx]

Supplementary Materials for

A Universal Model of Electrochemical Safety Limits *in* *vivo* for Electrophysiological Stimulation

# Supplementary Data

## Size and Bias Dependence of the Electrochemical Impedance

To illustrate some of the functional dependences of impedance that directly impact the current limits in equation (6) in the main manuscript, we use a modified Randles cell model and plot the different model parameters as a function of diameter and bias. Fig. S1(A) shows the dependence of the series resistance of the electrode contact as a function of the contact diameter and inter-contact separation. We see that the series impedance, comprising of the impedance of the electrical connections to the electrode (metal leads on the electrode and wires on the measurement equipment) and the spreading resistance in the media, rises exponentially with decreasing contact diameter. This is consistent with our observation of the exponential increase of G with decreasing contact diameter (Fig. 5(E) and (F)), as the effects of current crowding become more severe on smaller contacts with a smaller volume for current flow in the media. However, as discussed before, the series resistance does not impact the safety thresholds.

Because typical stimulation paradigms use square pulses of varying pulse widths (Lee et al., 2011), the impedance values at a single frequency of stimulation are unlikely to capture all the effects of the variation of electrochemical on the performance of the electrodes, which will cause the measured and predicted values to deviate for pulse widths significantly longer or shorter than those modeled here at 10kHz.

At higher frequencies, the double layer impedance dominates the charge injection mechanism at the interface, and the charge transfer process is mostly capacitive. Fig. S1(B) shows the variation of the double layer impedance (*Z_dl_*) with the contact diameter. We see an area dependence with a nearly exponential rise in the observed double layer impedance as we reduce the contact area. The double layer impedance can be expressed as:

$\boldsymbol{Z}_{\boldsymbol{dl}}\boldsymbol{=}\frac{\boldsymbol{1}}{\boldsymbol{C}_{\boldsymbol{dl}}}\left( \boldsymbol{j\omega} \right)^{\boldsymbol{-}\boldsymbol{\alpha}_{\boldsymbol{dl}}}$ **(S1)**

Therefore, *Z_dl_* increases as *C_dl_* decreases. The double layer impedance typically dominates the charge injection process for lower biases, and therefore directly the charge injection capacity of the electrode reduces with the contact diameter. The non-ideal frequency response of the double layer capacitance has been attributed to the surface roughness and heterogeneity of the surface of the electrode. At smaller diameters, the observed non linearities increase as the averaging effects over the contact area decrease, and the calculated value of *α_dl_* reduces, as shown in Fig.
S1(E).

The second mechanism of charge injection at the electrode interface is the faradaic transfer of ionic species at the electrode surface. At equilibrium, the charge injection process occurs primarily through the electrical double layer, and the impedance of the Faradaic branch is significantly higher. The variation in the Faradaic impedance follows similar trends as that of the double layer impedance, with a nearly exponential decrease observed with the contact diameter, although this dependence becomes weaker at larger contact sizes (Fig.S1(C)). A similar effect is also seen for the associated charge transfer resistance of the contact, which behaves similar to the faradaic impedance (Fig. S1(D)). As the bias on the electrode deviates from equilibrium, the faradaic impedance drops sharply, whereas the double layer capacitance does not vary significantly (Fig. S1(F)). This results in a gradual transition of the charge injection process from predominantly capacitive to predominantly Faradaic. The reduction in the electrochemical impedance at the interface leads to the non-linear voltage transients observed at large values of the injected current. This complex interplay of the interface elements makes it essential to account for the impact of the variations in the EIS spectra of the electrode, based on the electrode design and the media in which the electrode is placed, especially when comparing the electrode performance in an *in vivo* setup against benchtop, on the current safety limits during stimulation (Grill and Thomas Mortimer, 1995).

# Supplementary Figures and Tables

## Supplementary Figures


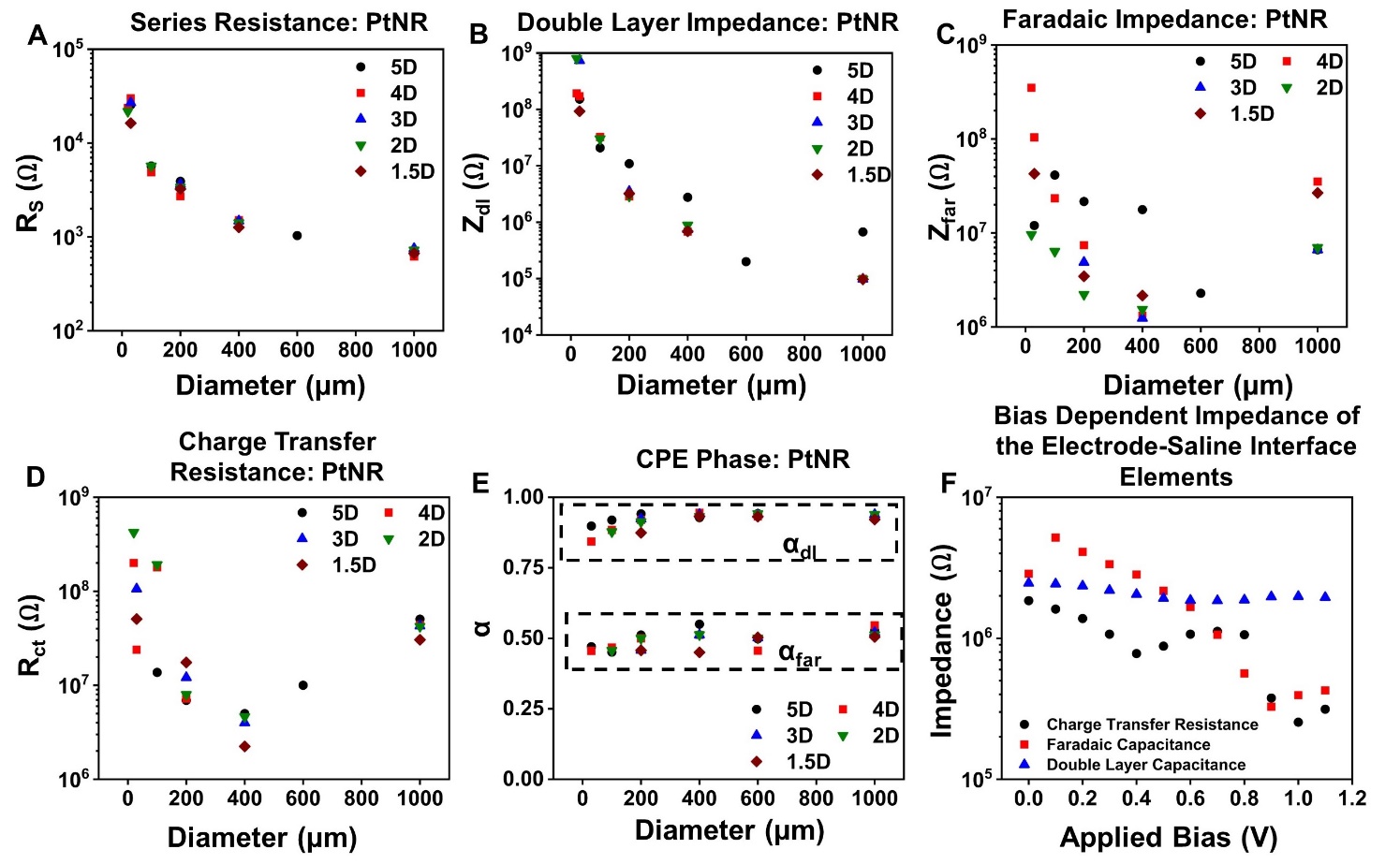


**Figure S1.** Variation of the PtNR contact circuit parameters as a function of diameter and for different inter-contact spacing for **(A)** series resistance (*R_S_*), **(B)** double layer reactance (*Z_dl_*), **(C)** Faradaic impedance (*Z_far_*), **(D)** charge transfer resistance (*R_ct_*), **(E)** CPE phase factor for the double-layer (*α_dl_*), and Faradaic (*α_far_*) elements. **(F)** Variation of the double layer and Faradaic branch impedance elements as a function of applied DC bias for a PtNR contact with 200µm diameter.


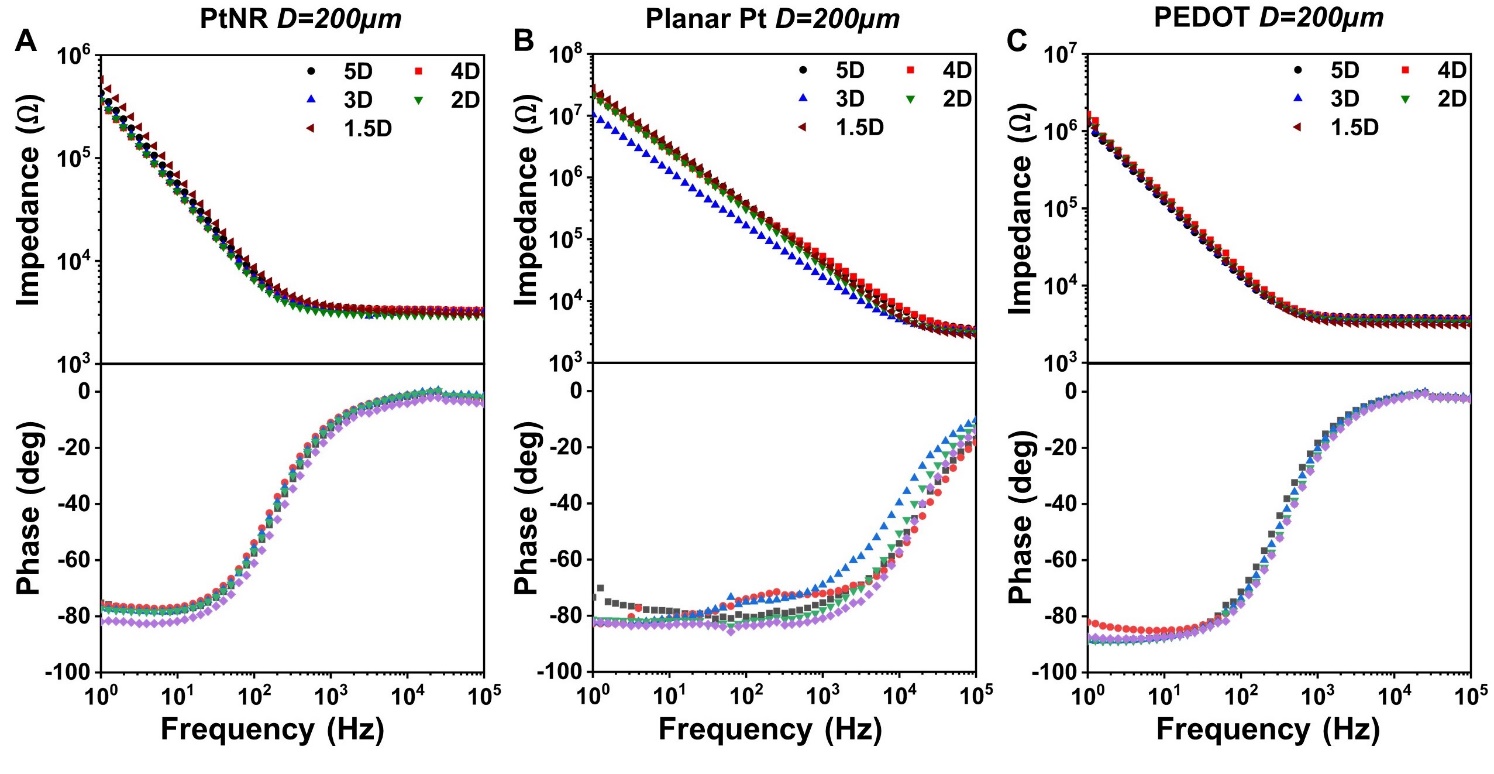
**Figure S2:** The variation of the impedance as a function of the inter-contact spacing for 200µm contacts, for **(A)** PtNR **(B)** Planar Pt and **(C)** PEDOT.


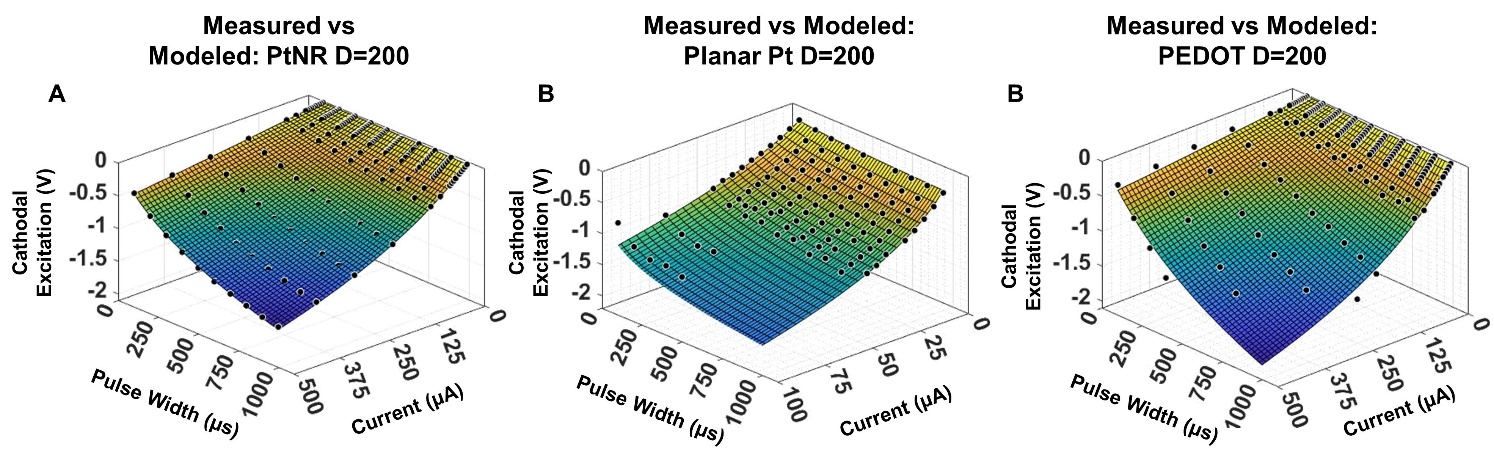


**Figure S3:** Fit for the cathodal excitation as a function of the pulse width and current, for **(A)** PtNR **(B)** Planar Pt and **(C)** PEDOT.


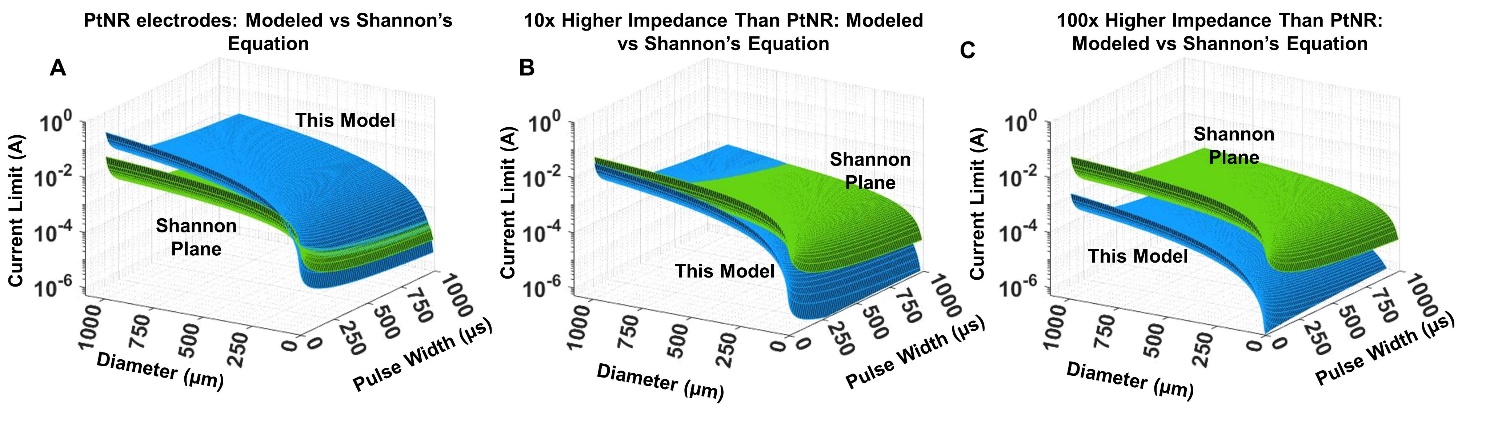


**Figure S4:** The variation of the current safety thresholds predicted by this model by varying the electrochemical impedance of the electrode-media interface. This effect is not captured in the Shannon’s equation. **(A)** The modeled data based on PtNR electrodes. **(B)** Modeled data based on theoretical electrode with 10 times higher impedance than the PtNR electrodes. **(C)** Modeled data based on theoretical electrode with 100 times higher impedance than the PtNR electrodes.

## Supplementary Tables

**Table S1.** Extracted circuit parameters for the PtNR electrodes as a function of the contact size and inter-contact separation

| **D(µm)** | **S** | **Material** | $\boldsymbol{R}_{\boldsymbol{S}}\boldsymbol{(\Omega)}$ | $\boldsymbol{C}_{\boldsymbol{DL}}\boldsymbol{(F)}$ | $\boldsymbol{\alpha}_{\boldsymbol{DL}}$ | $\boldsymbol{C}_{\boldsymbol{F}}\boldsymbol{(F)}$ | $\alpha_{F}$ | $\boldsymbol{R}_{\boldsymbol{CT}}\boldsymbol{(\Omega)}$ |
| --- | --- | --- | --- | --- | --- | --- | --- | --- |
| 1000 | 5D | PtNR | 6.49x10^2^ | 1.49x10^-6^ | 0.935 | 1.51x10^-7^ | 0.528 | 5.05x10^7^ |
| 1000 | 4D | PtNR | 6.18x10^2^ | 1.01x10^-6^ | 0.937 | 2.8410^-8^ | 0.546 | 4.37x10^7^ |
| 1000 | 3D | PtNR | 7.53x10^2^ | 1.02x10^-6^ | 0.939 | 1.51x10^-7^ | 0.521 | 4.28x10^7^ |
| 1000 | 2D | PtNR | 7.23x10^2^ | 1.02x10^-6^ | 0.939 | 1.43x10^-7^ | 0.512 | 4.23x10^7^ |
| 1000 | 1.5D | PtNR | 6.69x10^2^ | 1.03x10^-6^ | 0.9212 | 3.75x10^-8^ | 0.505 | 3.05x10^7^ |
| 400 | 5D | PtNR | 1.50x10^3^ | 3.62x10^-7^ | 0.9278 | 5.64x10^-8^ | 0.55 | 5.00x10^6^ |
| 400 | 4D | PtNR | 1.51x10^3^ | 1.47x10^-6^ | 0.932 | 7.60x10^-7^ | 0.456 | 1.03x10^6^ |
| 400 | 3D | PtNR | 1.48x10^3^ | 1.35x10^-6^ | 0.9383 | 8.06x10^-7^ | 0.5022 | 3.99x10^6^ |
| 400 | 2D | PtNR | 1.41x10^3^ | 1.13x10^-6^ | 0.9419 | 6.48x10^-7^ | 0.5008 | 4.62x10^6^ |
| 400 | 1.5D | PtNR | 1.26x10^3^ | 1.45x10^-6^ | 0.9316 | 4.62x10^-7^ | 0.503 | 2.24x10^6^ |
| 200 | 5D | PtNR | 3.91x10^3^ | 9.1410^-8^ | 0.941 | 4.63x10^-8^ | 0.5115 | 6.91x10^6^ |
| 200 | 4D | PtNR | 2.71x10^3^ | 3.51x10^-7^ | 0.9447 | 1.35x10^-7^ | 0.512 | 7.22x10^6^ |
| 200 | 3D | PtNR | 3.63x10^3^ | 2.85x10^-7^ | 0.9389 | 2.05x10^-7^ | 0.512 | 1.21x10^7^ |
| 200 | 2D | PtNR | 3.39x10^3^ | 3.44x10^-7^ | 0.932 | 4.50x10^-7^ | 0.513 | 7.97x10^6^ |
| 200 | 1.5D | PtNR | 3.24x10^3^ | 3.11x10^-7^ | 0.933 | 2.89x10^-7^ | 0.450 | 1.75x10^7^ |
| 100 | 5D | PtNR | 5.69x10^3^ | 4.80x10^-8^ | 0.919 | 2.42x10^-7^ | 0.451 | 1.37x10^7^ |
| 100 | 4D | PtNR | 4.89x10^3^ | 3.10x10^-8^ | 0.920 | 4.28x10^-8^ | 0.5 | 1.80x10^8^ |
| 100 | 3D | PtNR | 5.69x10^3^ | 3.33x10^-8^ | 0.913 | 1.57x10^-7^ | 0.5 | 1.92x10^8^ |
| 30 | 5D | PtNR | 2.55x10^4^ | 6.59x10^-9^ | 0.898 | 8.31x10^-5^ | 0.47 | 3.87x10^11^ |
| 30 | 4D | PtNR | 2.99x10^4^ | 5.86x10^-9^ | 0.884 | 9.57x10^-9^ | 0.467 | 2.39x10^7^ |
| 30 | 3D | PtNR | 2.70x10^4^ | 1.36x10^-9^ | 0.923 | 7.51x10^-10^ | 0.458 | 1.06x10^8^ |
| 30 | 1.5D | PtNR | 1.63x10^4^ | 1.08x10^-8^ | 0.874 | 2.34x10^-8^ | 0.457 | 5.07x10^7^ |

**Table S2.** Exponential parameters modeling the variation of the cathodal excitation as a function of the injected current, pulse width and electrochemical impedance in benchtop.

| $\mathbf{D}$ **(µm)** | **S** | **Material** | $\boldsymbol{k}_{\boldsymbol{2}}$ | $\boldsymbol{k}_{\boldsymbol{4}}$ |
| --- | --- | --- | --- | --- |
| 1000 | 5D | PtNR | 1.039 | 0.933 |
| 1000 | 4D | PtNR | 1.003 | 0.875 |
| 1000 | 3D | PtNR | 1.006 | 0.808 |
| 1000 | 2D | PtNR | 1.001 | 0.875 |
| 1000 | 1.5D | PtNR | 1.007 | 0.807 |
| 400 | 5D | PtNR | 1.021 | 0.927 |
| 400 | 4D | PtNR | 1.032 | 0.912 |
| 400 | 3D | PtNR | 1.049 | 0.951 |
| 400 | 2D | PtNR | 1.050 | 0.951 |
| 400 | 1.5D | PtNR | 1.038 | 0.921 |
| 200 | 5D | PtNR | 1.005 | 0.781 |
| 200 | 4D | PtNR | 1.077 | 0.919 |
| 200 | 3D | PtNR | 1.094 | 0.917 |
| 200 | 2D | PtNR | 1.085 | 0.899 |
| 200 | 1.5D | PtNR | 1.067 | 0.907 |
| 100 | 5D | PtNR | 1.057 | 0.809 |
| 100 | 4D | PtNR | 0.992 | 0.885 |
| 100 | 3D | PtNR | 0.978 | 0.945 |
| 100 | 2D | PtNR | 1.028 | 0.868 |
| 100 | 1.5D | PtNR | 0.967 | 0.895 |
| 50 | 5D | PtNR | 1.122 | 0.889 |
| 50 | 1.5D | PtNR | 1.139 | 0.897 |
| 50 | 2D | PtNR | 1.138 | 0.897 |
| 30 | 5D | PtNR | 1.122 | 0.990 |
| 30 | 4D | PtNR | 1.077 | 1.045 |
| 30 | 3D | PtNR | 1.061 | 1.067 |
| 30 | 2D | PtNR | 1.112 | 1.043 |
| 30 | 1.5D | PtNR | 1.118 | 0.871 |
| 1000 | 5D | Planar Pt | 0.945 | 0.758 |
| 1000 | 4D | Planar Pt | 0.951 | 0.799 |
| 1000 | 3D | Planar Pt | 0.9231 | 0.741 |
| 1000 | 2D | Planar Pt | 0.917 | 0.804 |
| 600 | 5D | Planar Pt | 0.845 | 0.620 |
| 600 | 4D | Planar Pt | 0.966 | 0.848 |
| 600 | 2D | Planar Pt | 0.936 | 0.871 |
| 400 | 5D | Planar Pt | 0.965 | 0.783 |
| 400 | 4D | Planar Pt | 0.965 | 0.783 |
| 400 | 3D | Planar Pt | 0.897 | 0.818 |
| 400 | 2D | Planar Pt | 0.892 | 0.823 |
| 400 | 1.5D | Planar Pt | 0.897 | 0.818 |
| 200 | 5D | Planar Pt | 0.752 | 0.597 |
| 200 | 4D | Planar Pt | 0.777 | 0.721 |
| 200 | 3D | Planar Pt | 0.793 | 0.636 |
| 200 | 2D | Planar Pt | 0.834 | 0.558 |
| 200 | 1.5D | Planar Pt | 0.749 | 0.529 |
| 100 | 5D | Planar Pt | 0.761 | 0.647 |
| 100 | 4D | Planar Pt | 0.648 | 0.550 |
| 100 | 2D | Planar Pt | 0.762 | 0.536 |
| 200 | 5D | PEDOT | 1.072 | 1.149 |
| 200 | 4D | PEDOT | 1.097 | 1.241 |
| 200 | 3D | PEDOT | 1.152 | 1.121 |
| 200 | 2D | PEDOT | 1.101 | 1.211 |
| 200 | 1.5D | PEDOT | 1.116 | 1.226 |
| 100 | 5D | PEDOT | 1.211 | 1.189 |
| 100 | 4D | PEDOT | 1.198 | 1.176 |
| 100 | 3D | PEDOT | 1.201 | 1.174 |
| 100 | 2D | PEDOT | 1.219 | 1.195 |
| 100 | 1.5D | PEDOT | 1.206 | 1.187 |
